# Three Rare Structural Anomalies: Right Aberrant Subclavian Artery, *Kommerell's Diverticulum*, and Isolated Left Vertebral Artery All Associated with Type B Aortic Dissection

**DOI:** 10.1155/2019/7927613

**Published:** 2019-03-17

**Authors:** Yasser Farag Elghoneimy, Medhat Reda Nashy, Ahmed Elsayed Mahmoud, Asayel Ali Alruwaili, Assayl Rabea Alotaibi

**Affiliations:** Department of Cardiothoracic Surgery, King Fahd Hospital of the University, Imam Abdulrahman Bin Faisal University, Al-Khobar, Saudi Arabia

## Abstract

**Introduction and Background:**

Right aberrant subclavian artery accounts for 0.5-1.8% of the population as the most frequently encountered aortic arch anomaly, while the prevalence of an isolated left vertebral artery ranges from 3 to 8%. Despite the low prevalence and the asymptomatic presentation of these structural anomalies, the development of cardiovascular complications and aneurysmal formation could happen as in Kommerell's diverticulum in a complicated right aberrant subclavian artery, which can undergo aneurysmal degeneration and dissection. Depending on the severity and the degree of the symptoms, the management of the patient can be determined.

**Case Presentation:**

A 51-year-old male hypertensive Pakistani patient was admitted complaining of chest and back pain; a CT of the aorta was done and showed type B aortic dissection associated with a right aberrant subclavian artery with an isolated left vertebral artery. A thoracic endovascular aneurysmal repair was done, and the patient improved afterward.

**Conclusion:**

The prevalence of these structural anomalies, the right aberrant subclavian artery, Kommerell's diverticulum, and isolated left vertebral artery with type B aortic dissection, is uncommon. Therefore, the earlier the diagnosis, the better the treatment. This is the first case report explaining the occurrence of these vascular anomalies together in Saudi Arabia.

## 1. Introduction

Aberrant right subclavian artery (ARSA) accounts for 0.5-1.8% of the population as the most frequently encountered aortic arch anomaly [1], while the prevalence of an isolated left vertebral artery (ILVA) ranges from 3 to 8% [2].

The ARSA originates directly from the aorta after the left subclavian artery, from the fourth branch of the aorta as its own, mostly crossing the midline posterior to the esophagus [3].

While the anomalies of the vertebral artery origins are various, they most commonly occur in the left than right and more unilateral than bilateral, usually located between the subclavian arteries and the left common carotid [4, 5].

*Kommerell's diverticulum* (KD) is a bullous figure which can arise from a complicated right or left aberrant subclavian artery; however, type B aortic dissection (TBAD) occurs when the tear is in the descending aorta which also might extend to the abdominal aorta [1, 6]. The most important structural anomalies that can determine the type of management are the presence of an aneurysm in addition to KD [1, 6]. Despite the low prevalence and the asymptomatic presentation of these structural anomalies, the development of cardiovascular complications and aneurysmal formation could happen as in KD in a complicated ARSA, which can undergo aneurysmal degeneration and dissection [1]. The most common symptoms are pressure like symptoms including dysphagia and thoracic pain usually in complicated ARSA [1], while vertigo and neck pain can occur in complicated ILVA [7]. Preoperative diagnosis includes different imaging modalities, such as computed tomography angiography (CTA) and magnetic resonance imaging. In ARSA, a barium swallow can also be used but not recommended in tracheoesophageal abnormalities. Hence, depending on the severity and the degree of the symptoms, the management of the patient can be determined.

## 2. Case Presentation

A 51-year-old hypertensive Pakistani male patient was admitted in the cardiac intensive care unit in King Fahd University Hospital on 30 July 2017 complaining of chest and back pain for two weeks prior to the presentation; he took nonsteroidal anti-inflammatory drugs but were not effective. Physical examination was done in the emergency department and revealed stable vital signs; the patient was conscious, moving all his limbs; there were warm palpable pulses of the upper arms.

**Figure 1:**
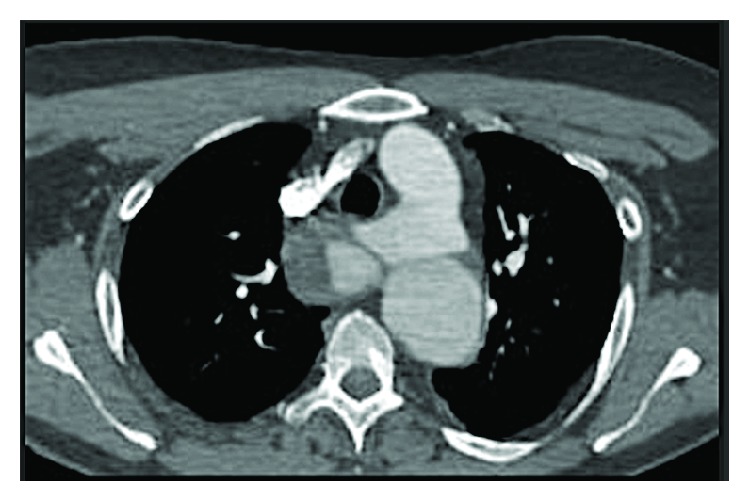
Preoperative chest CT showing type B aortic dissection from the origin of the left subclavian artery.

**Figure 2:**
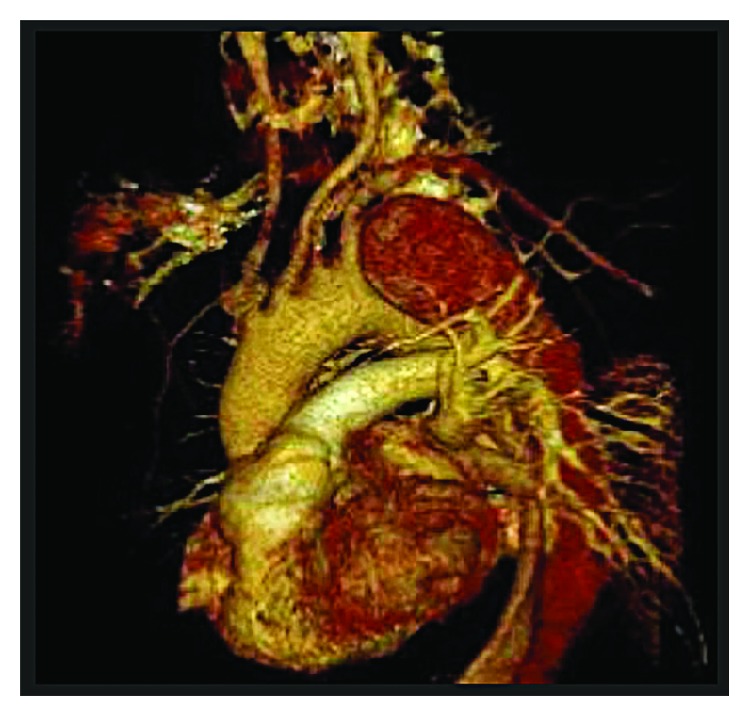
Preoperative virtual 3D CT angiogram of the aorta showing the separate origin of the left vertebral artery from the aortic arch.

The CTA of the aorta revealed a large dissection flap from the origin of the left subclavian artery extending down all the way to the level of renal arteries; there was enlargement of the false lumen at the proximal aorta with large aneurysmal dilatation (mural thrombus) and a compression of the true lumen (ascending aorta). Additionally, there was a large entry point seen 2 cm distal to the right subclavian artery. The aberrant right subclavian artery was noted crossing posterior to the trachea and arising as a last branch of the aortic arch distal to the left subclavian artery; also, the left vertebral artery was arising directly from the aortic arch. There was no other evidence of vascular dissection or occlusion below the level of the renal arteries (Figures 1 and 2).

**Figure 3:**
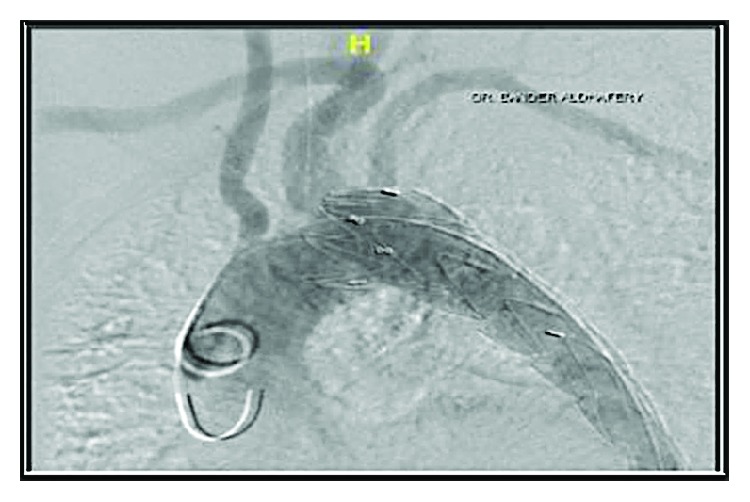
Intraoperative angiogram after stent placement.

The patient underwent general anesthesia for thoracic endovascular repair (TEVAR) two days after his first day of admission; the procedure was done through the right femoral approach; an angiogram was performed intraoperatively (Figure 3). Stent graft with a size of 34 *mm* × 15 *cm* was used and deployed into the descending thoracic aorta; the intimal entry tear was completely covered by the stent, and the false lumen was obliterated. A postdeployment angiogram revealed successful proximal occlusion of the entry point with no perfusion of the false lumen and good flow through both carotid and left vertebral arteries as well as patent right and left subclavian arteries.

**Figure 4:**
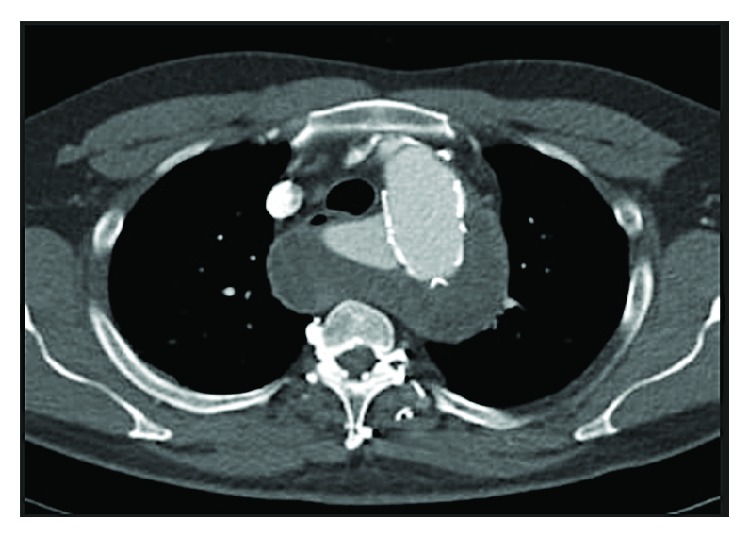
Postoperative axial CT angiography.

**Figure 5:**
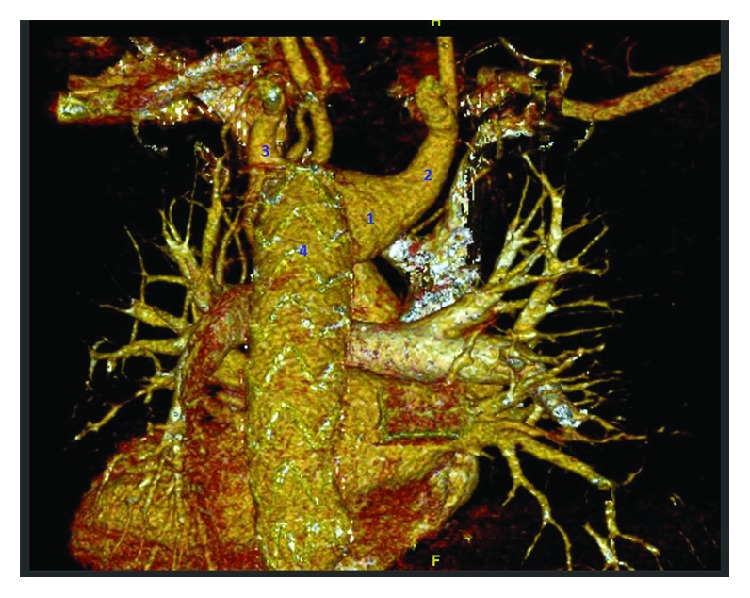
Postoperative CT aorta 3D reconstruction posterior view showing Kommerell's diverticulum (1), patent right aberrant subclavian artery (2), left subclavian artery (3), and a stent inside the descending aorta (4).

The patient was postoperatively moving all his limbs, and the peripheral pulses were intact. He got discharged one day after the surgery and was doing well afterward. We evaluated patient's prognosis by following up for 3 to 6 months for a chest X-ray, CTA, and CT aorta 3D reconstruction (Figures 4 and 5) which confirmed no endovascular leak as well as no ischemic or stroke signs in the clinical follow-up; we assessed the patency of the subclavian arteries by examining the bilateral upper arms' blood supply in addition to radial arteries by evaluating the pulses with no evidence of morbidity.

## 3. Discussion

The first case that reported the occurrence of an aortic dissection with an aberrant right subclavian artery was by DeBakey et al. in 1955 [8, 9]. Our case has the combination of aortic dissection, ARSA, and KD with the involvement of ILVA.

Moreover, the first case that reported three vascular anomalies together of dissecting aortic aneurysm, ARSA, and ILVA was in 1996 [9].

Regarding the management of these anomalies, if KD and aortic aneurysm were absent, the ARSA could be treated with the transposition of the right subclavian artery to the right common carotid artery through a right supraclavicular incision, dissection, and transaction of the artery passing over to the left side of the esophagus [10, 11]. The ILVA can be reconstructed and reimplanted during an aortic arch replacement and open stent-grafting technique [12].

There is no standard surgical repair for KD; however, its size and the presence of persisting symptoms can determine its surgical treatment [12].

The surgery is considered when the diameter of the diverticulum is more than 30 mm, and/or the diameter size of the descending aorta adjacent to KD is more than 50 mm [12], while TEVAR is recommended in complicated TBAD. Complicated TBAD with inadequate proximal landing zones and with ILVA is considered a challenging issue in TEVAR [4]. Therefore, TEVAR through an open surgical repair is not preferred if it was associated with aortic arch pathologies, although a surgical approach using a stented elephant trunk technique as an alternative to TEVAR in patients with inadequate proximal fixation zones revealed favorable outcomes [4].

## 4. Conclusion

The prevalence of these structural anomalies, the right aberrant subclavian artery, Kommerell's diverticulum, and isolated left vertebral artery with type B aortic dissection, is uncommon. Therefore, the earlier the management, the better the outcome. This is the first case report explaining the occurrence of these vascular anomalies together in Saudi Arabia.
